# The Key Factors Predicting Dementia in Individuals With Alzheimer’s Disease-Type Pathology

**DOI:** 10.3389/fnagi.2022.831967

**Published:** 2022-04-25

**Authors:** Andrew N. McCorkindale, Ellis Patrick, James A. Duce, Boris Guennewig, Greg T. Sutherland

**Affiliations:** ^1^Charles Perkins Centre and School of Medical Sciences, Faculty of Health and Medicine, The University of Sydney, Sydney, NSW, Australia; ^2^School of Mathematics and Statistics, Faculty of Science, The University of Sydney, Sydney, NSW, Australia; ^3^The ALBORADA Drug Discovery Institute, Cambridge Biomedical Campus, University of Cambridge, Cambridge, United Kingdom; ^4^Brain and Mind Centre and School of Medical Sciences, Faculty of Medicine and Health, The University of Sydney, Sydney, NSW, Australia

**Keywords:** Alzheimer’s disease, cognition, machine learning, transcriptomics, pathology

## Abstract

Dementia affects millions of individuals worldwide, yet there are no effective treatments. Alzheimer’s disease, the most common form of dementia, is characterized by amyloid and tau pathology with amyloid accumulation thought to precipitate tau pathology, neurodegeneration, and dementia. The Religious Orders Study and Memory and Aging Project (ROSMAP) cohort is a unique resource with quantitative pathology from multiple brain regions, RNA sequencing, and longitudinal cognitive data. Our previous work applying machine learning to the RNA sequencing data identified lactoferrin (LTF) as the gene most predictive of amyloid accumulation with a potential amyloidogenic mechanism identified *in vitro* and with cell-culture models. In the present study, we examined which pathologies and genes were related to cognitive status (dementia, mild impairment, and no cognitive impairment) and rate of cognitive decline. Tau load in the anterior cingulate and *ADAMTS2*, encoding a metallopeptidase, were the respective regional pathology and gene most associated with cognitive decline, while *PRTN3*, encoding a serine protease, was the key protective feature. *ADAMTS2*, but not *PRTN3*, was related to amyloid and tau load in the previous study while *LTF* was not related to cognitive decline here. These findings confirm a general relationship between tau pathology and dementia, show the specific importance of tau pathology in the anterior cingulate cortex and identify ADAMTS2 as a potential target for slowing cognitive decline.

## Introduction

Dementia currently affects 50 million people worldwide and is projected to affect 152 million by 2050 due to an aging population (World Health Organisation, 2017). The most common form of dementia is Alzheimer’s disease (AD), but despite extensive research and multiple clinical trials, there are still no disease-modifying therapies. Further target validation with greater mechanistic understanding is urgently required to identify new therapeutic opportunities. Multi-omics studies of human *post-mortem* brain tissue and RNA sequencing (RNA-seq), in particular, promise much in understanding neurodegenerative mechanisms, but the data is complex, and the key findings reported from various studies to date have mainly been discordant.

One of the challenges in using human data is that elderly donors each have a unique combination of lifestyle, medical history, and neuropathology. Thus, larger cohort sizes with both clinical and biological information are required. The Religious Orders Study and Memory and Aging Project (ROSMAP) are two combined longitudinal studies that have recruited ∼3400 nuns, priests, brothers (ROS), and laypersons (MAP) and have carried out ∼1500 brain autopsies (De Jager et al., 2018). This autopsy data has shown the presence of 236 different combinations of pathology in the ROSMAP cohort, showing that most elderly people have multiple pathologies present and that these do not follow a fixed pattern (Boyle et al., 2018). In addition, the contribution of each pathology to cognitive impairment varies between individuals with AD pathology, accounting for approximately 50% of cognitive decline overall but ranging between 22 and 100% for an individual (Boyle et al., 2018). The same group also found that only 41% of the variation in cognitive decline could be explained by neuropathology (Boyle et al., 2013) and that ∼33% of dementia cases could not be directly attributed to any neuropathology (Boyle et al., 2019). These studies also showed that some individuals with substantial AD pathology did not have cognitive decline.

Previous neuropathology studies in the ROSMAP cohort have only used a binary AD diagnosis variable in their analyses despite available quantitative tau and amyloid data from multiple brain regions (Boyle et al., 2018). Instead, we have included quantitative regional AD pathology data to find whether pathology in a particular brain region drives cognitive decline. Furthermore, while previous studies have employed parametric linear models, the relationship between AD pathology and cognitive decline may not be linear (e.g., amyloid may accumulate until a certain threshold before triggering a sudden acceleration of tau pathology). We thus apply non-parametric machine learning algorithms to the ROSMAP quantitative neuropathology data to identify the regional pathology most related to cognitive decline.

The ROSMAP bulk tissue RNA-seq dataset includes over 600 samples and has been used in numerous studies (Felsky et al., 2018; Mostafavi et al., 2018; Olah et al., 2018; Tsatsanis et al., 2021). However, the data was first processed a decade ago using the sequence aligner Bowtie which has been superseded (Dobin et al., 2013; Baruzzo et al., 2017). In addition, most of the past studies have used parametric methods such as differential expression with few applying machine learning (ML). While ML is commonly used for classification with a focus on metrics such as accuracy, other powerful methods (Wenric and Shemirani, 2018) have been explicitly developed for feature selection (e.g., identifying the genes that best differentiate AD from controls). Boruta is a robust feature selection method used to identify all of the input variables that are “important” in predicting an outcome such as AD diagnosis or cognitive score (Kursa and Rudnicki, 2010; Kursa, 2014). Boruta works by introducing “shadow features” (randomly shuffled copies of the input variables) and then comparing the performance of the true values of the input variables with these “shadow features” in a classification or regression model (random forest used here). It runs iteratively and outputs a list of the input variables ranked by Z-score showing the input variables that consistently outperformed the highest ranked shadow feature.

We previously re-processed the ROSMAP data through an updated pipeline and applied Boruta to the ROSMAP cohort to identify genes related to AD pathology (Tsatsanis et al., 2021). We identified *lactotransferrin* (*LTF*) as a key predictor of AD status and amyloid pathology load before placing a direct interaction between lactoferrin (encoded by lactotransferrin) and β-amyloid precursor protein (APP) *in vitro* with a potential mechanism for lactoferrin to promote amyloidogenic processing of APP and β-amyloid accumulation shown in cell-based models. We also ran differential expression analysis where *LTF* was ranked 2360th. The low ranking in the differential expression compared to Boruta combined with the laboratory validation of lactoferrin indicates that Boruta may detect genes overlooked by differential expression. In the previous study, we focused on the genes related only to AD pathology. Therefore, in the current study, we have again applied ML to the same ROSMAP cohort to identify the key genes and pathologies associated with the clinical diagnosis of dementia and the rate of cognitive decline.

## Materials and Methods

This study was undertaken following permission from the Rush Alzheimer’s Disease Center and data accessed *via* the AD Knowledge portal. ROSMAP comprises two active longitudinal cohort studies that have recruited individuals aged 65 and over with no known dementia in order to gather extensive clinical information prior to a comprehensive neuropathological examination at death. RNA-seq data from the dorsolateral prefrontal cortex was available for 638 subjects (De Jager et al., 2018). As 98.4% of the cases were from a non-Hispanic White background, the remaining 1.6% was excluded in order to reduce a source of genetic variation. The cohort of 589 from our previous study was reduced to 542 to include only individuals with complete cognitive data.

The subjects were first divided based on their final consensus clinical diagnoses irrespective of their AD pathology diagnosis. There were 172 (39% AD pathology) with no cognitive impairment (NCI), 147 (54% AD pathology) with mild cognitive impairment (MCI), and 223 (82% AD pathology) with dementia. Second, the subjects were divided based on their slope of episodic memory decline and slope of global cognitive decline. These variables represent the “estimated person-specific rate of change” in the cognition variables over time. It comes from a linear mixed-effects model, which controls for age at baseline, sex, and years of education (Wilson et al., 2015). The 100 subjects with the slowest decline were compared to the 100 subjects with the fastest decline irrespective of their clinical diagnoses.

### Tissue Preparation and RNA-Sequencing

Detailed methods of the RNA-seq data preparation have been published previously (Mostafavi et al., 2018). Briefly, RNA-seq libraries were prepared using the strand-specific dUTP method (polyA selection) with sequencing performed on the Illumina HiSeq with 101-bp paired-end reads and coverage of 50 million reads. The input fastq files for the present study were downloaded from https://www.synapse.org/#!Synapse:syn8612097. These fastq files had been reverted from binary alignment matrix (BAM) files produced when the samples had been first aligned using Bowtie. The BAM files included the mapped and unmapped reads from the original alignment. These fastq files were re-processed using our custom pipeline as follows: quality of the input data was assessed using FastQC (version 0.11.3) before the reads were mapped to the GRCh38.p10 reference genome using the STAR (version 2.5.2a) aligner and known GENCODE genes were quantified using RSEM (version 1.3.0).

RSEM count data were imported into the R project environment, and analysis was performed using the methods described previously (Tsatsanis et al., 2021). Reads with primarily zero counts were filtered out, leaving 20,494 genes for downstream analyses. Outlier samples were identified using principal components analysis and hierarchical clustering. Reads were normalized using the edgeR package with the trimmed mean of means method (TMM). A principal components analysis showed that there was a clear batch effect (data not shown). Counts were log-transformed prior to the removal of the batch effect using the ComBat algorithm (Johnson et al., 2007). These batch-corrected counts were used as the input for the machine learning algorithms.

### Boruta

Boruta was run over 10,000 iterations in R (version 4.0.2) for both classification and regression on the RNA-seq and pathology data. The classification runs were performed to identify the input variable relevant to clinical diagnosis (dementia vs. NCI, dementia vs. MCI, and MCI vs. NCI) and fast decline vs. slow decline of episodic memory and global cognition. All analyses were balanced by taking a random sample of the larger group. The regression runs were performed to identify the input variables relevant to the slope of global cognitive decline, the slope of episodic memory decline, and subsequently the genes associated with *ADAMTS2* and *PRTN3*.

### Machine Learning Confirmation and Validation

The stability of the Boruta results was confirmed by running three algorithms (“ranger,” “xgbTree,” and “glmnet”) using the “caret” R package (Kuhn, 2008). All algorithms were run with repeated k-fold cross-validation (five folds and 20 repeats) as the sampling method. The ‘‘varImp’’ function from the ‘‘caret’’ package was then used to extract the feature importance from each model to compare to the Boruta results. The transcripts of interest were validated using the bulk RNA-seq and control single-cell RNA-seq from the AD consensus website^1^ as well as a single-cell analysis of the ROSMAP data (Mathys et al., 2019; Morabito et al., 2020). The bulk RNA data include the ROSMAP data used here as well as the Mount Sinai Brain Bank (Wang et al., 2018) and Mayo Clinic Studies (Allen et al., 2016).

### Neuropathology Data

The neuropathology analyses were done using the wider ROSMAP cohort to maximize the available sample size (∼1300 with autopsy data at the time of application). Quantitative neuropathology methods have been previously published (Bennett et al., 2003; Mostafavi et al., 2018) and include cortical density of abnormally phosphorylated tau (eight brain regions), areal fraction of Aβ (eight brain regions), and counts of neuritic plaques, diffuse plaques, and neurofibrillary tangles in five brain regions. All pathology variables are defined and can be requested *via* the RADC Research Resource Sharing Hub^2^. Only subjects with complete neuropathology data across all brain regions were used. This resulted in 713 subjects with complete data for 62 variables being used as the input for the analysis (Variable descriptions and missing data summarized in Supplementary Table 4). Results were confirmed using the same algorithms and sampling methods described above for the RNA-seq data with the addition of a simple decision tree model run using the “rpart” algorithm. All analysis code can be found at: https://github.com/binfnstats/ML_cognition.

## Results

### RNA-Sequencing Analysis

The Boruta feature selection algorithm was applied to the ROSMAP bulk tissue RNA-seq data to identify the genes predictive of cognitive status. There were 46 genes for dementia vs. NCI, 22 for dementia vs. MCI, and six for MCI vs. NCI (complete lists in Supplementary Table 1). Boruta ranks the input variables by Z-score with the top ten genes for dementia vs. NCI and dementia vs. MCI shown in Table 1. Two genes (*PRTN3* and *PPDPF*) were downregulated in dementia vs. NCI or MCI.

**TABLE 1:** Top 10 genes for classifying the subjects into dementia and no cognitive impairment and for classifying subjects into mild cognitive impairment and dementia.

Dementia vs. no			Dementia vs. mild		
cognitive impairment			cognitive impairment		
Gene symbol	*z*-score	Direction*	Gene symbol	*z*-score	Direction*
PRTN3	7.51	Down	PRPF4	7.59	Down
SLC4A11	6.60	Up	ADAMTS2	5.82	Up
MEIS3	6.06	Down	RPSAP15	5.81	Down
AGPAT1	6.05	Down	PRTN3	5.33	Down
GSEC	5.93	Down	KIF5A	5.13	Up
PPDPF	5.82	Down	TACR2	4.95	Down
ARHGEF34P	5.58	Up	BRD1	4.94	Up
TCP10L	5.51	Down	OVCH1-AS1	4.78	Down
IL15	5.49	Up	ANKRD30B	4.48	Down
NUMBL	4.90	Down	NPPA	4.36	Down

*Higher or lower mean expression in dementia.

Next, we explored whether there were genes that were associated with participants exhibiting a more rapid decline in cognition: particularly of episodic memory. One hundred subjects with the fastest decline in episodic memory were compared to the same number of subjects with the slowest decline using Boruta. There were 63 genes that differentiated between these two groups with *PRTN3*, *ADAMTS2*, and *PPM1D* ranked highest (Top ten in Table 2). From a similar analysis on subjects with the fastest and slowest global cognitive decline (average across the five cognitive domains assessed in the ROSMAP study), there were 21 genes common to both lists (with *PRTN3* and *ADAMTS2* again ranked highly) (Complete gene lists in Supplementary Table 1) including seven that were common to dementia vs. NCI analysis (*PRTN3, GSEC, FAM160A2, ANKRD19P, SLC6A9, SLC4A11*, and *AQP6*).

**TABLE 2:** Top 10 genes for classifying the subjects into fast episodic memory decline and slow episodic memory decline and top 10 genes predictive of episodic memory in the full cohort.

Gene symbol	*z*-score	Direction*	Gene symbol	*z*-score	Cor.*
PRTN3	7.47	Down	PRTN3	7.45	+
ADAMTS2	6.22	Up	ADAMTS2	6.83	−
PPM1D	5.92	Up	ZCCHC10	6.43	+
ENSG00000216895	5.3	Down	TAF1D	6.3	+
LINC02172	5.18	Up	CCN4	5.76	−
SLC6A9	4.95	Up	ZNF334	5.59	+
TMEM72-AS1	4.88	Down	HMGN2	5.48	+
PPP4R1	4.8	Up	PPP4R1	5.47	−
QDPR	4.57	Up	SLC4A11	4.99	−
ZNF334	4.56	Down	ANKRD19P	4.96	+

*Higher or lower mean expression in dementia.

*Pearson correlation between gene and slope of episodic memory decline.

As a follow-up, we tested whether there were genes that predicted the slope of episodic memory decline across the entire cohort. 38 genes predicted the progression of episodic memory decline, with 23 of these genes also associated with the fast decliners group (Supplementary Table 1). *PRTN3* and *ADAMTS2* were again the top-ranked genes along with several transporter genes (*SLC6A9, SLC38A2, SLC4A11, AQP6*).

*ADAMTS2* and *PRTN3* were consistently ranked highly in the Boruta analyses. They are both linked to peripheral immune response and extracellular matrix maintenance (summarized in Supplementary Table 2) but there is little information on their role in the human brain. Therefore, a gene association analysis was done using Boruta to find the genes predictive of *ADAMTS2* and *PRTN3* levels using all 20493 other genes as input. There were 205 genes predictive of *ADAMTS2* and 98 genes predictive of *PRTN3* levels (Supplementary Table 3), although there were no overlaps between the two “co-expressed” gene lists or in the STRING protein-protein interaction database (Supplementary Figures 1A,B). The genes that were predictive of *PRTN3* included genes associated with calcium-signaling, such as *HPCAL1*, and synaptic function. The genes predictive of ADAMTS2 levels included genes associated with glucocorticoid response (e.g., *HSD11B2*, *ELK1*, *PTPRU*) and neuropeptide signaling (e.g., *VGF, SORT1, IRS1*) (Hofer et al., 2008; Gutièrrez-Mecinas et al., 2011).

### Confirmation and Validation of Target Genes

The high rankings of *ADAMTS2* and *PRTN3* in the Boruta analysis were confirmed using other ML algorithms. The dementia vs. NCI and fast vs. slow analyses of global cognitive and episodic memory decline were repeated with three other algorithms (“ranger,” “xgbTree,” and “glmnet”) using repeated cross-fold validation to see if the target genes were identified by other ML methods. PRTN3 was ranked 1st by the tree-based algorithms (“xgbTree” and “ranger”) in all analyses while (Supplementary Table 4). ADAMTS2 was ranked between 3rd and 6th by the tree-based algorithms for episodic memory and global cognitive decline. Both genes were ranked lower by the linear “glmnet” algorithm.

*ADAMTS2* and *PRTN3* were validated using the AD consensus transcriptomics online resource (see text footnote 1) (Morabito et al., 2020). *PRTN3* was lower in symptomatic AD (AD pathology diagnosis and dementia) compared to controls and asymptomatic AD (AD pathology diagnosis without dementia) in the Mayo Clinic temporal cortex data (not present in the Mount Sinai Brain Bank analysis). *ADAMTS2* levels were consistently higher in symptomatic AD relative to both controls and asymptomatic AD (no difference between controls and asymptomatic AD) in all datasets (Supplementary Figures 1C,D).

Two single-cell RNA-seq datasets were used to identify the cell types producing the transcripts of these two genes. The first dataset included controls only (aged 67–90) and it showed that *PRTN3* and *ADAMTS2* transcripts were mainly expressed by neurons with lower expression in other cell types (microglial expression of ADAMTS2 only) (Supplementary Figures 1E,F). The second dataset included ROSMAP controls and AD subjects (*n* = 48) (Mathys et al., 2019), and it showed that *PRTN3* was only expressed by excitatory and inhibitory neurons and was downregulated in AD. *ADAMTS2* was expressed in and upregulated in AD in all cell types (Supplementary Table 5).

### Neuropathology Analysis

The ROSMAP neuropathology data includes various indices of Aβ and tau load across multiple brain regions as well as other neuropathologies and demographic information. Following exclusions for missing data, 62 variables from 713 individuals were used as the input to Boruta (Supplementary Table 6). 34 variables were identified for classifying dementia vs. NCI, 28 for classifying dementia from MCI, and six for classifying MCI vs. NCI, with the top 10 variables for each analysis shown in Table 3. Tau pathology variables were consistently ranked higher than amyloid variables across all analyses, with tangles in the anterior cingulate cortex (ACC) ranked highest each time.

**TABLE 3:** Top 10 pathology variables predictive of clinical diagnosis.

Rank	Dementia vs. NCI	*z*-score	Dementia vs. MCI	*z*-score	MCI vs. NCI	*z*-score
1	AT8 ACC	17.74	AT8 ACC	8.98	AT8 ACC	13.61
2	AT8 Total	12.12	AT8 InfTemp	8.34	NFT Total	6.29
3	NFT Total	11.86	NP MidTemp	7.95	AT8 InfTemp	4.92
4	AT8 InfTemp	11.85	AT8 MidFront	7.54	AT8 Hip	4.08
5	AT8 MidFront	11.42	AT8 Angular	7.36	NFT MidTemp	3.70
6	AT8 Angular	9.49	AT8 SupFront	7.12	NFT EntoCort	3.67
7	AT8 Hip	9.46	AT8 Total	6.51		
8	AT8 SupFront	9.04	TDP stage	5.87		
9	NFT MidTemp	8.32	Parkinson’s Dx	5.20		
10	NP MidTemp	8.15	CERAD score	5.16		

*AT8, Abnormally phosphorylated tau from immunohistochemistry; NFT, neurofibrillary tangles from silver staining; NP, neuritic plaques from silver staining; CERAD, Consortium to establish a registry for Alzheimer’s disease score; TDP, Tar-DNA Binding Protein stage, full variable description in Supplementary Table 4.*

As with the RNA-seq analysis, the pathology cohort was split into the 100 subjects with the fastest or slowest rates of cognitive decline. There were 27 variables predictive of accelerated episodic memory decline and 26 for accelerated global cognitive decline (Top 10 shown in Table 4 with a full list in Supplementary Table 6). Tau pathology was again the main factor, with eight of the top 10 variables for each analysis being tau related. Tangles in the ACC were ranked highest in both analyses, while age was also in the top five for both. The analysis was repeated across the full cohort for potential associations with the slope of episodic memory decline and global cognitive decline. 44 variables were found to be important for the progressive loss of episodic memory and global cognition, with 43 of these common to both. Tau tangles in the ACC was ranked highest, with the same pathology in the middle and superior frontal gyri also being in the top five factors. Age, a diagnosis of Parkinson’s disease, and middle temporal neuritic plaques were also features in the top 10.

**TABLE 4:** Top 10 variables for separating the 100 subjects with the fastest cognitive decline from the 100 subjects with the slowest cognitive decline.

Rank	Episodic decline	*z*-score	Global decline	*z*-score
1	AT8 ACC	16.28	AT8 ACC	15.20
2	AT8 Total	11.68	AT8 Total	11.36
3	Age	10.65	AT8 SupFront	10.94
4	NFT Total	10.34	Age	10.13
5	AT8 Angular	9.25	AT8 MidFront	9.79
6	AT8 MidFront	8.88	NFT Total	8.16
7	AT8 InfTemp	8.82	NFT CA1Hip	7.86
8	NFT CA1Hip	8.46	AT8 Angular	7.78
9	NP MidTemp	8.41	NP MidTemp	7.73
10	AT8 SupFront	7.75	AT8 InfTemp	7.54

*AT8, Abnormally phosphorylated tau from immunohistochemistry; NFT, neurofibrillary tangles from silver staining; NP, neuritic plaques from silver staining, full variable description in Supplementary Table 4.*

### Confirmation of the Neuropathology Analysis

Three alternative ML algorithms were used to confirm the Boruta neuropathology results above. Tangles in the ACC was again the highest-ranked feature in the “xgbTree” and “ranger” models for all analyses (Supplementary Table 7). AD pathology measures were ranked lower by the linear “glmnet” algorithm, and hippocampal sclerosis, a previous head injury as well as a Parkinson’s disease diagnosis were also ranked highly across the three types of analyses. A simple decision tree model (“rpart”) showed that tangles in the ACC alone could be used to classify the subjects into fast episodic and slow episodic memory decline (AUC = 0.78), and to a lesser extent, dementia and NCI (AUC = 0.73).

## Discussion

As the previous ML analysis with Boruta using ROSMAP cohort data identified the gene encoding lactotransferrin or lactoferrin (*LTF*) as a key contributor to AD pathology (Tsatsanis et al., 2021), the aim of this study was to identify the key genes and pathologies contributing to cognitive decline. Transcriptomic analysis suggested a role for several genes in cognitive decline with *ADAMTS2* and *PRTN3* featuring prominently, while tau pathology in the ACC was the most predictive neuropathological index from the ROSMAP neuropathology data. *PRTN3* levels decreased, while *ADAMTS2* increased with cognitive decline. There was no overlap in co-expressed gene lists suggesting that they have independent effects on dementia pathogenesis.

*ADAMTS2* encodes an extracellular matrix protein (a disintegrin and metalloproteinase with thrombospondin motifs, 2) that is mainly known for cleaving the propeptides of collagen type I and II. It has been linked to brain diseases such as schizophrenia and vascular dementia (Romay et al., 2019; Ruso-Julve et al., 2019) and was predictive of both tau and amyloid pathology in our previous analysis of the ROSMAP data (Tsatsanis et al., 2021). While our gene association analysis suggested that it is involved in stress response and neuropeptide signaling, there is not a clear mechanistic link to AD pathology. One possible mechanism for how *ADAMTS2* modifies AD risk could be through dysregulated extracellular matrices, and like *PRTN3*, affecting blood-brain-barrier integrity (Anwar et al., 2021). In a related role, *ADAMTS2* can cleave and inactivate reelin (Yamakage et al., 2019), an extracellular matrix protein that is important for synaptic plasticity (Bock and May, 2016; Lussier et al., 2016). Reelin and other extracellular matrix components are associated with susceptible neurons of the entorhinal cortex in AD (Santa-Maria et al., 2010) and reduced reelin is linked to increased AD pathology in animal models (Kocherhans et al., 2010). Reelin has also been linked to other brain diseases such as schizophrenia and frontotemporal dementia (Sáez-Valero et al., 2003; Negrón-Oyarzo et al., 2016). The association between *ADAMTS2* and reelin could explain ADAMTS2’s association with multiple brain diseases but further mechanistic studies are clearly required.

*PRTN3* encodes the neutrophil serine protease, proteinase-3 (aka myeloblastin). It is mainly expressed by neutrophils where it shares similar roles to the other neutrophil serine proteases (cathepsin G and neutrophil elastase). PRTN3 is constitutively expressed on neutrophils membrane where a key function is to restore vascular integrity following infection (Korkmaz et al., 2010; Kuckleburg and Newman, 2013). There is little information about *PRTN3* in the brain, but it is known to act on protease-activated receptor (PAR)1 and PAR2 (Kuckleburg and Newman, 2013) which are important mediators of synaptic transmission (Price et al., 2021). *PRTN3* is mainly expressed by neurons (Mathys et al., 2019) and probably in the synaptic compartment given the Boruta results, which identified several associated synaptic genes. *PRTN3* was also in a module enriched for synapse-related genes in a previous ROSMAP analysis where it was also highly negatively correlated (31st of 13,484) with amyloid (Mostafavi et al., 2018). *PRTN3* was not in the Boruta results of our previous AD pathology study but this does not exclude a moderate association. Overall, this could mean that higher *PRTN3* levels are protective against amyloid pathology or that the lower levels of *PRTN3* in dementia are a sign of synaptic loss.

The pathology results here support our current understanding that tau pathology has a greater relationship with cognitive decline than amyloid pathology (Arriagada et al., 1992) and that AD pathology is the major pathology type related to cognitive decline (Boyle et al., 2013, 2018, 2019). Over two-thirds of the pathology variables were identified in at least one analysis, showing that many pathologies contribute to cognitive decline. However, tau pathology in the ACC was consistently the highest-ranked variable for predicting clinical diagnosis as well as the progression of cognitive decline. Tangles in the ACC was particularly powerful for differentiating MCI from NCI, suggesting that it is an important region in the onset of cognitive decline. Cortical tau pathology was also ranked higher than hippocampal and entorhinal tau pathology, indicating the importance of the neocortical spread in dementia.

Within the brain, the ACC is topographically situated above the corpus callosum and has roles in executive function and emotional regulation *via* neural pathways connecting to the frontal and temporal lobes. It is regarded as a transition zone between the allocortex, that includes the hippocampus, entorhinal cortex, and the overlying neocortex. It is significantly atrophied in AD but only develops amyloid pathology in Thal Phase 2 once the amyloid deposition has already spread throughout the neocortex (Thal et al., 2002; Jones et al., 2006). In the present study, neither amyloid nor tau pathology was particularly high in the ACC, with tau levels substantially lower than in the entorhinal cortex and hippocampus. More generally, tau-associated pathology is substantially lower in the neocortex than in the medial temporal regions, yet cognitive decline is strongly related to the spread of pathology to the neocortex [as in the Braak staging system (Braak and Braak, 1991)]. One explanation is that the ACC and neocortex are simply more vulnerable to tau pathology than the hippocampus. Another possibility is that the ACC and neocortex functionally compensate for the effects of tau pathology in the allocortex, but this renders it vulnerable to even small amounts of tau pathology that subsequently develop there. Answering the question of why the ACC appears to be vulnerable to the effects of tau pathology is vital to understanding the pathogenesis of dementia.

While this study provides insights into the mechanisms of dementia there are some notable limitations. There are challenges associated with human post-mortem brain tissue studies such as agonal effects and RNA degradation. However, the large size of the ROSMAP study and relatively high average RIN (∼7) increases the chance of identifying true disease effects here. A further limitation is that the subjects were split into fast cognitive decline and slow cognitive decline using the ROSMAP slope of cognitive decline data. Although this was the best available metric, we did not have the scores from each timepoint for each individual or the time interval between their final cognitive assessment and autopsy so the slope may not accurately reflect cognitive decline for some individuals. Lastly, the size and scope (quantitative regional neuropathology, longitudinal cognition, and RNA-sequencing) of the ROSMAP dataset means that there was no dataset available where we could replicate the machine learning approach used here. However, both *PRTN3* and *ADAMTS2* (as well as other highly ranked genes such as *PPDPF*, *SLC6A9*, *SLC4A11*) were recently identified as relevant to AD progression by an independent study using deep learning on the Mount Sinai Brain Bank and ROSMAP transcriptomic datasets (Wang et al., 2021).

## Conclusion

We have shown that tau pathology in the ACC and two genes (*PRTN3* and *ADAMTS2*) are strongly associated with the rate of cognitive decline in the ROSMAP cohort. There was overlap with our previous study where *ADAMTS2* was shown to be predictive of total tau and superior frontal amyloid (Tsatsanis et al., 2021). Notably, all three key transcripts from our two studies (*LTF*, *PRTN3*, and *ADAMTS2*) encode proteins associated with peripheral immune responses, but their functions in the brain are not well understood. While higher *PRTN3* may be protective, an interaction between immune genes, the amyloid-related *LTF*, and the tau-related *ADAMTS2* could provide insights into how amyloid accelerates tau pathology. Further research is needed to clarify the roles of these proteins in dementia associated with AD pathology.

## Data Availability Statement

The original contributions presented in the study are included in the article/Supplementary Material, further inquiries can be directed to the corresponding author.

## Author Contributions

AM, GS, and BG: conceptualization. AM and BG: methodology and validation. AM: formal analysis, investigation, resources, data curation, writing – original draft preparation, and visualization. GS and BG: supervision, project administration, and funding acquisition. All authors: writing – review and editing.

## Conflict of Interest

BG is a director of Pacific Analytics Pty. Ltd., and SMRTR Pty. Ltd., Australia, a founding member of the International Cerebral Palsy Genomics Consortium, and a member of the Australian Genomics Health Alliance. The remaining authors declare that the research was conducted in the absence of any commercial or financial relationships that could be construed as a potential conflict of interest.

## Publisher’s Note

All claims expressed in this article are solely those of the authors and do not necessarily represent those of their affiliated organizations, or those of the publisher, the editors and the reviewers. Any product that may be evaluated in this article, or claim that may be made by its manufacturer, is not guaranteed or endorsed by the publisher.

## Footnotes

1
https://swaruplab.bio.uci.edu/consensusAD

2
https://www.radc.rush.edu/

## Supplementary Material

Supplementary Table 1 Complete Boruta results from the RNA sequencing analyses.

Supplementary Table 2 Gene information and key functions of ADAMTS2 and PRTN3.

Supplementary Table 3 Complete gene lists from the Boruta gene association analyses of ADAMTS2 and PRTN3.

Supplementary Table 4 Top 50 genes from the algorithms used to confirm the results of the Boruta classification analyses.

Supplementary Table 6 Complete Boruta results from the pathology analyses.

Supplementary Table 7 Top 20 variables from the algorithms used to confirm the results of the Boruta pathology analyses.
