# Supplementary material for: The Key Factors Predicting Dementia in Individuals With Alzheimer’s Disease-Type Pathology
Source: Front Aging Neurosci. 2022 Apr 25;14:831967. doi: 10.3389/fnagi.2022.831967 (PMC9085578; doi:10.3389/fnagi.2022.831967)
Supplement: Supplementary Table 2 — Gene information and key functions of ADAMTS2 and PRTN3. [file Table_2.DOCX]

**Supplementary Table S2. Gene information and functions of ADAMTS2 and PRTN3.**

| **ADAMTS2** | **PRTN3** |
| --- | --- |
| - **Gene structure**: 22 exons located at 5q35.3 (179,110,852-179,345,460) (Colige, Nuytinck et al. 2004) - **Summary**: Encodes the 134 kDa “a disintegrin-like and metalloproteinase with thrombospondin type 1 motifs 2” (aka procollagen I N-Proteinase) protein. Main known function is to cleave the propeptides of type I and II collagen prior to fibril assembly. Secreted as a zymogen that is activated by furin. Has a Zn^2+^ binding site. (Colige, Nuytinck et al. 2004) - **Known disease association**: ADAMTS2 variants cause Ehlers-Danlos syndrome, dermatosparaxis type which is characterised by extreme skin fragility (Colige, Nuytinck et al. 2004) | - **Gene structure**: five exons located at 19p13.3 (840,964-848,174) (Sturrock, Franklin et al. 1992) - **Summary**: Encodes a 28 kDa serine protease enzyme (proteinase-3 aka myeloblastin) that is expressed mainly in neutrophils. Main known function is to degrade extracellular matrix components such as elastin and collagen but also has effects on protease-activated receptors (Rao, Wehner et al. 1991) - **Known disease association**: Major target antigen of antineutrophil cytoplasmic antibodies in Wegener’s granulomatosis (Brockmann, Schwarting et al. 2002) |
| **Broader and potential central nervous system functions of ADAMTS2** | **Broader and potential central nervous system functions of PRTN3** |
| - Involved in glucocorticoid response in macrophages (Hofer, Frankenberger et al. 2008) - Binds to other collagen types III, V, and VI (Bekhouche, Leduc et al. 2016) - Cleaves and inactivates reelin (Yamakage, Kato et al. 2019) as do other ADAMTS enzymes (Ogino, Hisanaga et al. 2017, Song and Dityatev 2018) - regulates blood vessel homeostasis and angiogenesis (Bekhouche and Colige 2015) - influences TGF-β signalling via DKK (Bekhouche, Leduc et al. 2016) - involved in clinical response to anti-psychotics in patients with schizophrenia (Ruso-Julve, Pombero et al. 2019) - Modulates cardiac hypertrophy by inhibiting activation of the phosphoinositide 3-kinase/protein kinase B (PI3K/AKT) signalling pathway (Wang, Chen et al. 2017) | - Enhances vascular endothelial cell barrier function and involved in neutrophil endothelial transmigration (Kuckleburg and Newman 2013) - Degrades the anti-inflammatory protein progranulin. Knockout of proteinase-3 led to higher progranulin levels and an impaired inflammatory response (Kessenbrock, Dau et al. 2011) - binds to protease-activated receptors (PAR) which are important in synaptic transmission (Price, Mercuri et al. 2021) - able to cause apoptosis via caspase-3 (Loison, Xu et al. 2014) - Can cleave protease-activated receptor 1 to block the activity of thrombin (Mihara, Ramachandran et al. 2013) - - stimulate mitogen-associated protein kinase activation via binding to PAR-1 (Mihara, Ramachandran et al. 2013) |
