# Supplementary material for: The Key Factors Predicting Dementia in Individuals With Alzheimer’s Disease-Type Pathology
Source: Front Aging Neurosci. 2022 Apr 25;14:831967. doi: 10.3389/fnagi.2022.831967 (PMC9085578; doi:10.3389/fnagi.2022.831967)
Supplement: Supplementary file 5 [file Table_5.DOCX]

**Supplementary Table S5. Mean expression of PRTN3 and ADAMTS2 across cell types from the Mathys *et al*. (2019) single cell RNA sequencing study (Ref).**

| **Mean expression in no pathology group** | | | | | | |
| --- | --- | --- | --- | --- | --- | --- |
| Gene | Excitatory | Inhibitory | Astrocyte | Oligo | Oligo precursor | Microglia |
| *PRTN3* | 0.011 | 0.002 | NA | NA | NA | NA |
| *ADAMTS2* | 0.088 | 0.017 | 0.009 | 0.009 | 0.005 | 0.007 |
|  | | | | | | |
| **Mean expression in AD pathology group** | | | | | | |
| Gene | Excitatory | Inhibitory | Astrocyte | Oligo | Oligo precursor | Microglia |
| *PRTN3* | 0.007 | 0.001 | NA | NA | NA | NA |
| *ADAMTS2* | 0.147 | 0.020 | 0.012 | 0.012 | 0.012 | 0.015 |
|  | | | | | | |
| **Fold change between AD pathology and no pathology group** | | | | | | |
| Gene | Excitatory | Inhibitory | Astrocyte | Oligo | Oligo precursor | Microglia |
| *PRTN3* | -0.730* | -0.954 | NA | NA | NA | NA |
| *ADAMTS2* | 0.745* | 0.278 | 0.334 | 0.365 | 1.268 | 0.994 |

Excitatory = Excitatory neuron, Inhibitory = Inhibitory neuron, Oligo = oligodendrocyte. * = p < 0.05
