# Supplementary material for: The Key Factors Predicting Dementia in Individuals With Alzheimer’s Disease-Type Pathology
Source: Front Aging Neurosci. 2022 Apr 25;14:831967. doi: 10.3389/fnagi.2022.831967 (PMC9085578; doi:10.3389/fnagi.2022.831967)
Supplement: Supplementary file 8 [file Data_Sheet_1.docx]

**Supplementary figures**


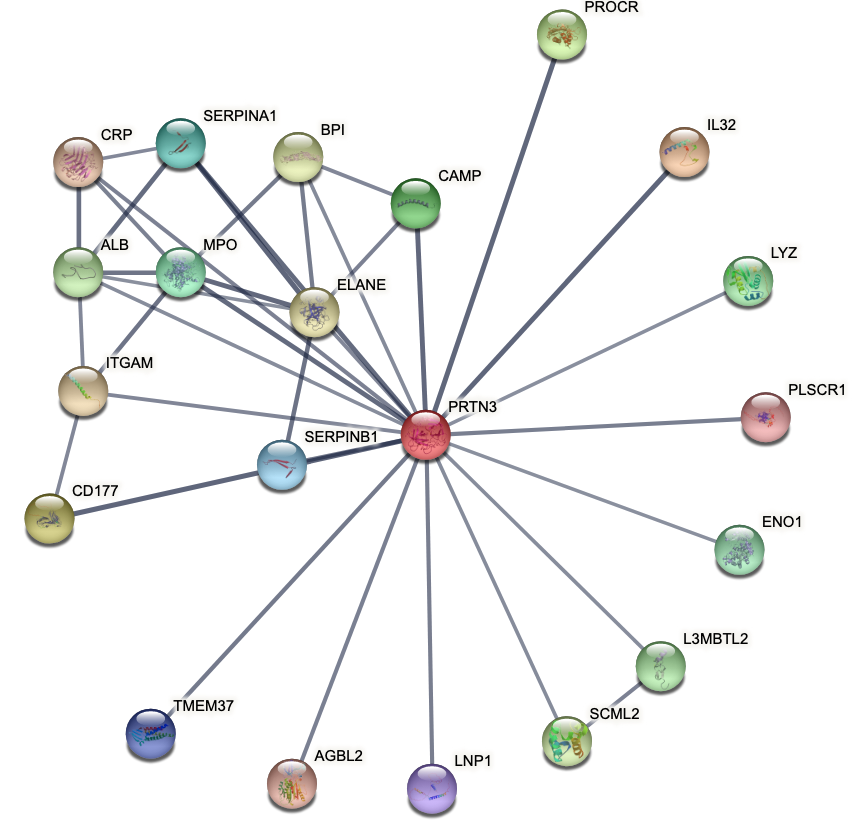


**Figure S1a. Top 20 protein-protein interactions for PRTN3**

A protein-protein network from the STRING database (Szklarczyk, Gable et al. 2018)for PRTN3. Thickness of line denotes strength of association. Colours have been arbitrarily assigned.


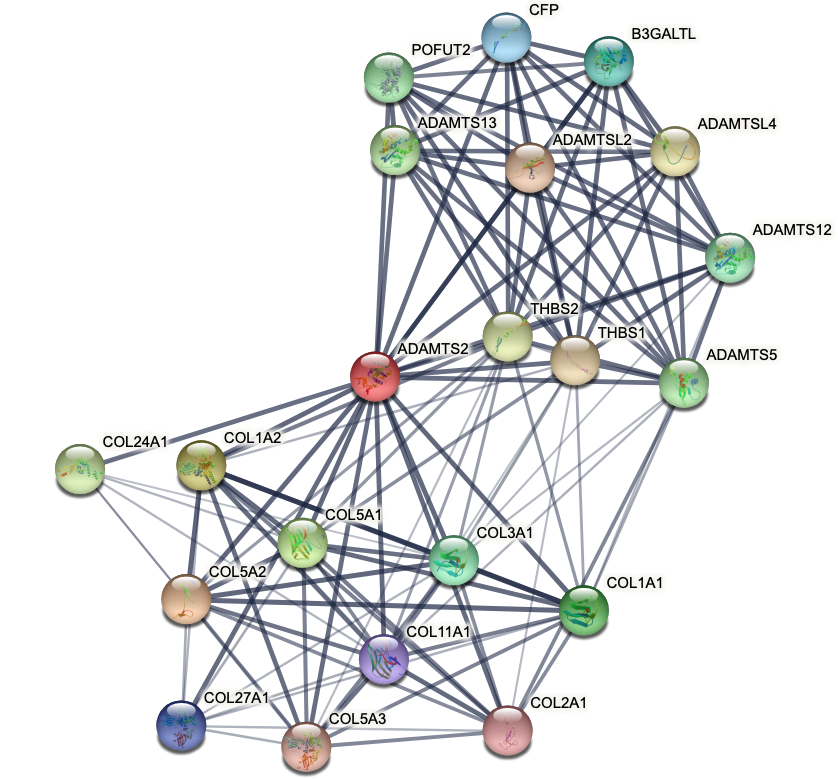


**Figure S1b. Top 20 protein-protein interactions for ADAMTS2**

A protein-protein network from the STRING database (Szklarczyk, Gable et al. 2018) for ADAMTS2. Thickness of line denotes strength of association. Colours have been arbitrarily assigned.

**A**

**B**

**Figure S1c. Expression of PRTN3 across two transcriptomic datasets.**

Box plots showing PTRN3 transcript expression in A. Mayo Clinic and B. ROSMAP post-mortem human brain bulk tissue transcriptomic data. AsymAD = subject met pathological criteria for AD without having cognitive impairment, AD = Subject met pathological criteria for AD and had cognitive impairment, Control = No pathology diagnosis of AD, Mayo.TC = Mayo Clinic Temporal cortex, ROSMAP.PFC= ROSMAP prefrontal cortex. Group comparisons made with the Wilcoxon Ranked-Sum test. *PRTN3* not expressed in Mt Sinai data set. Both plots generated using the AD Consensus Transcriptomics online resource **(**<https://swaruplab.bio.uci.edu/consensusAD>)

**A**

**C**

**B**

**D**

**F**

**E**

**Figure S1d. Expression of *ADAMTS2* across multiple transcriptomic datasets.** Box Plots show the expression of *ADAMTS2* transcripts across the six Accelerating Medicines Partnership for Alzheimer’s disease (AMP-AD) datasets (A-F). AsymAD = subject met pathological criteria for AD without having cognitive impairment, AD = Subject met pathological criteria for AD and had cognitive impairment, Control = No pathology diagnosis of AD, Mayo.TC = Mayo Clinic Temporal cortex, ROSMAP.PFC= ROSMAP prefrontal cortex. Group comparisons made with the Wilcoxon Ranked-Sum test. All plots generated using the AD Consensus Transcriptomics online resource **(**<https://swaruplab.bio.uci.edu/consensusAD>).

**Figure S1e. Expression of *PRTN3* in single-cell RNA-seq controls**


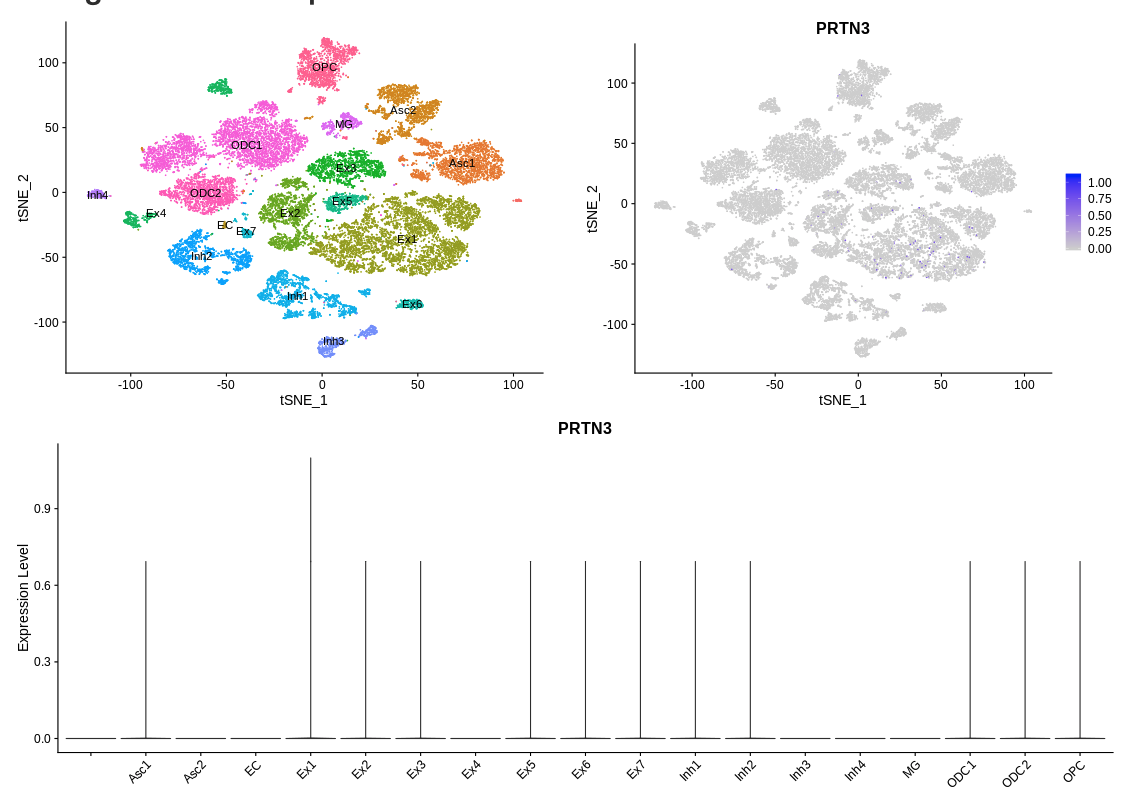


**C**

**A**

**B**

A. A t-distributed stochastic neighbor embedded (tSNE) plot shows the clustering of different cell types in the Morabito *et al.* single cell RNA-seq data of human post-mortem prefrontal cortex from neurologically normal controls (Morabito, Miyoshi et al. 2020) B. A tSNE plot shows *PRTN3* transcript levels in each cell cluster from A and C. A violin plot of the (low) *PRTN3* transcripts. Asc= Astrocyte, EC = endothelial cell, Ex = excitatory neuron, Inh = Inhibitory neuron, MG = microglia, ODC = Oligodendrocyte, OPC = Oligodendrocyte precursor cells. All plots generated using AD Consensus Transcriptomics tools **(**<https://swaruplab.bio.uci.edu/consensusAD>).


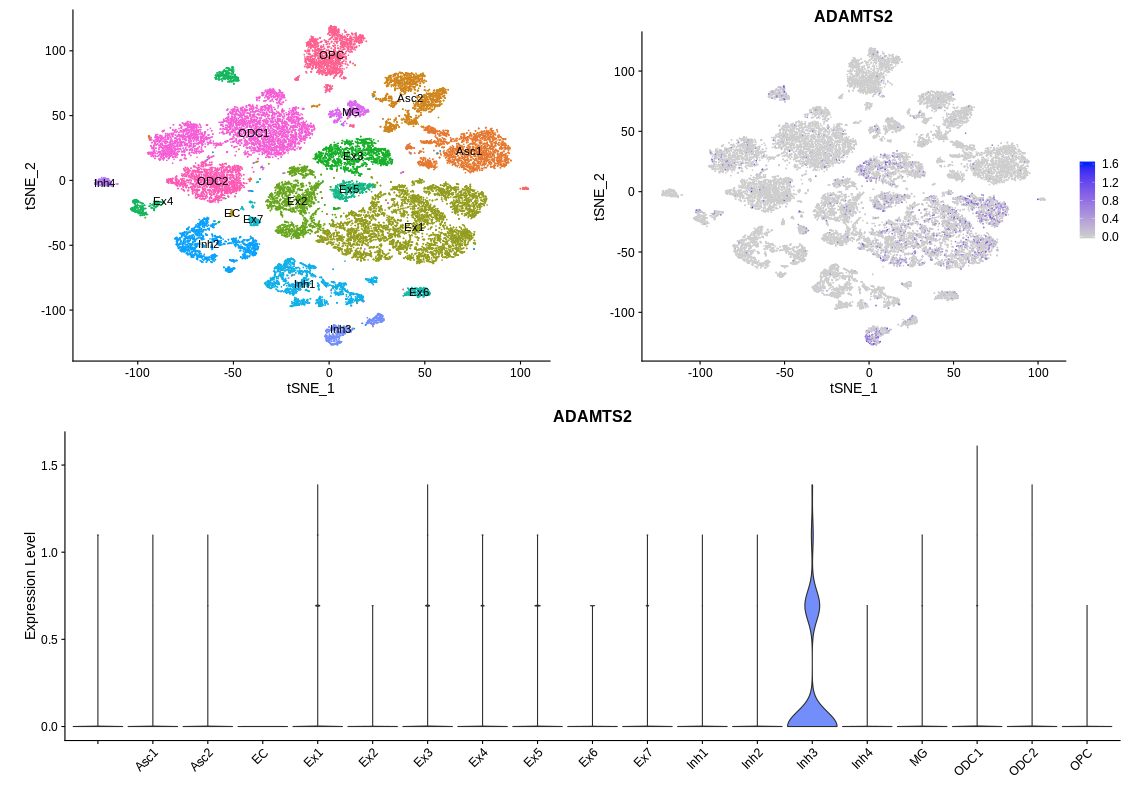


**A**


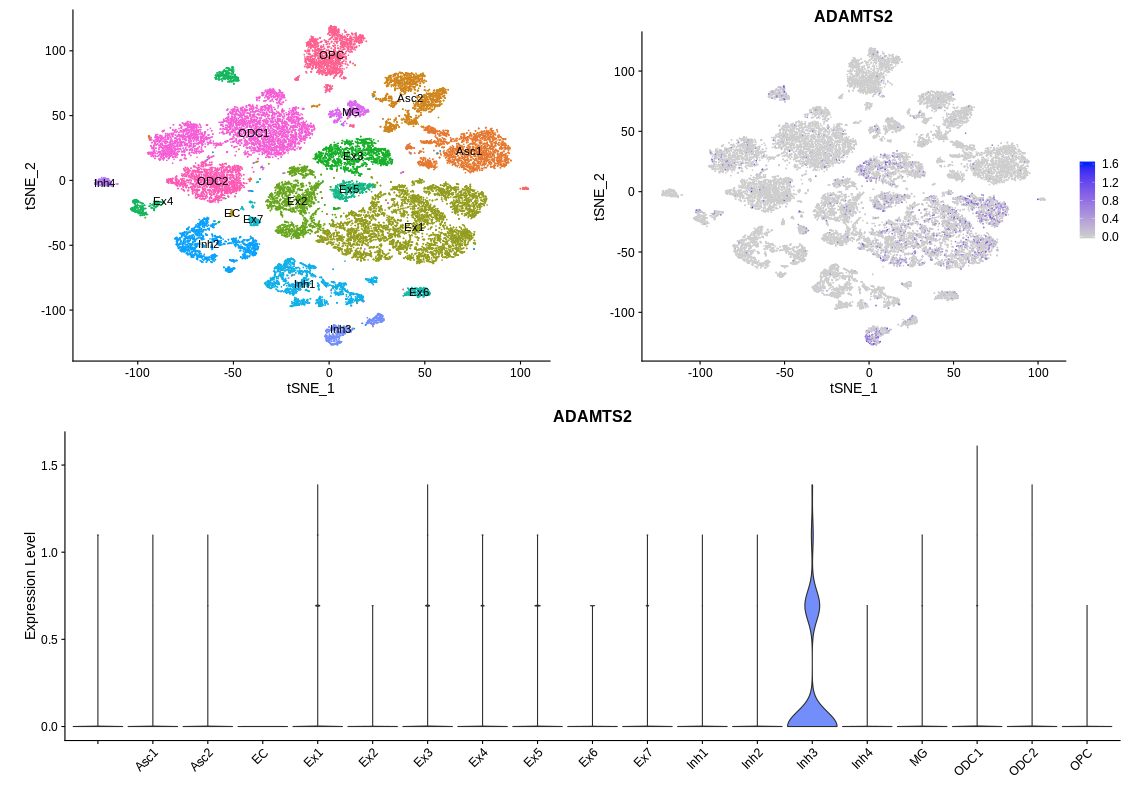


**B**

**C**

**Figure S1f. Expression of *ADAMTS2* in single-cell RNA-seq controls.**

A. A t-distributed stochastic neighbor embedded (tSNE) plot shows the clustering of different cell types in the Morabito *et al.* single cell RNA-seq data of human post-mortem prefrontal cortex from neurologically normal controls (Morabito, Miyoshi et al. 2020) B. A tSNE plot shows *ADAMTS2* transcript levels in each cell cluster from A and C. A violin plot of the (low) *ADAMTS2* transcripts. Asc= Astrocyte, EC = endothelial cell, Ex = excitatory neuron, Inh = Inhibitory neuron, MG = microglia, ODC = Oligodendrocyte, OPC = Oligodendrocyte precursor cells. All plots generated using AD Consensus Transcriptomics tools **(**<https://swaruplab.bio.uci.edu/consensusAD>).

**References**

Morabito, S., E. Miyoshi, N. Michael and V. Swarup (2020). "Integrative genomics approach identifies conserved transcriptomic networks in Alzheimer’s disease." Human Molecular Genetics **29**(17): 2899-2919.

Szklarczyk, D., A. L. Gable, D. Lyon, A. Junge, S. Wyder, J. Huerta-Cepas, M. Simonovic, N. T. Doncheva, J. H. Morris, P. Bork, L. J. Jensen and Christian v. Mering (2018). "STRING v11: protein–protein association networks with increased coverage, supporting functional discovery in genome-wide experimental datasets." Nucleic Acids Research **47**(D1): D607-D613.
